# Molecular and histopathological characterization of seminoma patients with highly elevated human chorionic gonadotropin levels in the serum

**DOI:** 10.1007/s00428-023-03698-0

**Published:** 2023-12-14

**Authors:** Christoph Seidel, Finn-Ole Paulsen, Tim Nestler, Richard Cathomas, Marcus Hentrich, Pia Paffenholz, Carsten Bokemeyer, Axel Heidenreich, Daniel Nettersheim, Felix Bremmer

**Affiliations:** 1 Department of Oncology, Hematology and Stem Cell Transplantation With Division of Pneumology, University Medical Center Hamburg-Eppendorf, Martinistraße 52, 20246 Hamburg, Germany; 2 Department of Urology, Federal Armed Forces Hospital Koblenz, Koblenz, Germany; 3 Division of Oncology/Hematology, Kantonsspital Graubünden, Chur, Switzerland; 4 Red Cross Hospital Munich, Ludwig Maximilian University of Munich, Munich, Germany; 5 Department of Urology, Uro-Oncology, Robot-Assisted and Specialized Urologic Surgery, University of Cologne Faculty of Medicine and University Hospital Cologne, Cologne, Germany; 6 Department of Urology, Medical University Vienna, Vienna, Austria; 7 Department of Urology, Urological Research Laboratory, Translational UroOncology, Medical Faculty and University Hospital Düsseldorf, Heinrich Heine University Düsseldorf, Düsseldorf, Germany; 8 Institute of Pathology, University Medical Center, Göttingen, Germany

**Keywords:** Germ cell tumor, Seminoma, HCG, Testicular cancer

## Abstract

Approximately 30% of seminoma (SEM) patients present with moderately elevated human chorionic gonadotropin (hCG) levels at first diagnosis. In case of high hCG serum levels, the presence of a non-SEM component, i.e. choriocarcinoma (CC), may be assumed. To characterize cases described as pure seminoma with high serum hCG levels, tissue samples and DNA were analyzed. Patient files from an international registry were screened for patients with SEM and extraordinarily high hCG serum levels. IHC and qRT-PCR analysis was performed for markers of SEM, embryonal carcinoma (EC) and CC/trophoblast cells. The cell lines TCam-2 (SEM), 2102EP, NCCIT, NT2/D1 (EC) and JAR, JEG3 and BeWo (CC) were included for comparison. Of 1031 SEM patients screened, 39 patients (3.7%) showed hCG serum levels > 1000 U/l. Of these, tumor material for IHC and RNA for qRT-PCR was available from *n* = 7 patients and* n* = 3 patients, respectively. Median pre-orchiectomy serum hCG level was 5356 U/l (range: 1224–40909 U/L). Histopathologically, all investigated samples were classified as SEM with syncytiotrophoblast sub-populations. SEM cells were SALL4^+^ / OCT3/4^+^ / D2-40^+^, while syncytiotrophoblast cells were hCG^+^ / GATA3^+^ / p63^+^ and SOX2^−^/CDX2^−^. qRT-PCR analysis detected trophoblast stem cell markers CDX2, EOMES and TFAP2C as well as the trophectoderm-specifier TEAD4, but not GATA3. Additionally, SOX17 and PRAME, but not SOX2, were detected, confirming the pure SEM-like gene expression signature of the analyzed samples. In conclusion, excessively increased hCG serum levels can appear in patients with pure SEM. To explain detectable hCG serum levels, it is important to diagnose the subtype of a SEM with syncytiotrophoblasts.

## Introduction

Germ cell tumors (GCTs) comprise over 95% of testicular cancers and account for approximately 75.000 new cases and over 9.000 deaths worldwide each year [1]. Despite being a rare tumor entity, testicular cancer is the most common malignancy in male adolescents and young adults and its incidence has been increasing during recent years [2, 3]. As GCTs originate from an early developmental stage, they divide into seminomas (SEM) and non-seminomatous germ cell tumors (NSGCT). Characteristically, GCTs, especially non-seminomas (non-SEMs), harbor a broad phenotypic heterogeneity due to a differentiation potential based on pluripotency features of the stem cell-like population of embryonal carcinoma [4]. Both types originate from testicular germ cell neoplasia in situ (GCNIS), which is considered to originate from developmentally arrested primordial germ cells (PGC) [4]. SEMs resemble masses of GCNIS, appearing morphologically close to PGC and proliferate as homogeneous tumors, retaining features of germinal lineage. Approximately 30% of SEM patients with advanced disease present with moderately elevated serum levels of beta human chorionic gonadotropin (hCG). The possible presence of this diagnostic marker in SEM is well documented and accounted for the presence of syncytiotrophoblastic cells (STC) [5, 6]. However, in these cases, serum hCG levels rarely exceed 500 IU/L and extraordinarily high hCG levels have only been described in few cases [7, 8]. Therefore, in case of high hCG levels the presence of a non-SEM component in the tumor, i.e. choriocarcinoma (CC), may be suspected. To better characterize hCG^+^ SEM patients, we previously conducted an international registry with the German Testicular Cancer Study Group in collaboration with the Global Germ Cell Tumor Collaborative Group (G3) analyzing clinical features from 1031 patients with SEM and hCG levels above normal at first diagnosis according to local laboratory ranges [9]. A small subset of patients in this registry presented with extraordinarily high hCG levels at initial diagnosis, suggesting that this subgroup may represent a distinct prognostic category, as these patients had an unfavorable outcome. Recently, Wagner and colleagues described as an independent predictor for relapse [10]. Since this retrospective registry provided no central pathological review, the question was raised whether non-SEM components were present in the tumor specimens of these patients, therefore suggesting an incorrect diagnosis of classical SEM when NSGCT was present. A correct diagnosis and stratification of GCTs in SEM and NSGCT is important, because treatment and prognosis can differ for these entities [11]. Consequently, we conducted a comprehensive analysis of tissue and RNA material from patients from this registry diagnosed with SEM with excessively elevated serum hCG levels defined as > 1000 U/L. The aim of this study was to validate the diagnosis of pure SEM in patients and to find a rationale for the highly elevated hCG levels in these patients.

## Material and methods

### Study population

A registry composed of SEM cases from 19 centers or study groups on behalf of the Global Germ-Cell Cancer Cooperative Group (G3) (*n* = 1031) was screened for patients with exceptionally high hCG levels (arbitrarily defined as > 1000 U/L) in the serum at the time of first diagnosis, as described before [9]. Treating physicians were requested to provide tumor material for central pathological review. If available, formalin-fixed and paraffin embedded (FFPE) tissue was sent to the institute of pathology of the university medical center Göttingen, Germany, for further histomorphological and immunohistochemical (IHC) analyses. Here HE- and Immun-slides and FFPE blocks were available. All cases examined in the present study were processed in accordance with the EAU guidelines [12]. In case of availability of fresh frozen tissue isolated RNA samples were analyzed at the institute of molecular urology at the university medical center Düsseldorf, Germany. The study was approved by the ethics committees of the medical faculties of Göttingen and at the participating local institutions (no. 20/9/20).

### Cell lines

The GCT cell lines TCam-2 (SEM), 2102EP, NCCIT and NT2/D1 (embryonal carcinoma, EC) as well as JAR, JEG3 and BeWo (CC) were included and grown as described [13, 14] (Table 1). Cell lines are checked regularly for mycoplasma contaminations and STR analyses are available of each cell line upon request.

**Table 1:** The GCT cell lines TCam-2 (SEM), 2102EP, NCCIT and NT2/D1 (EC) as well as JAR, JEG3 and BeWo (CC) were included and grown as described [11, 12]

Cell line	Entity	Medium	Supplements	Kindly provided by
2102EP	Embryonal carcinoma	DMEM (1x) + GlutaMAX-I	10% FBS, 1% P/S (10,000 U), 1% L-Glutamin (200 mM)	Dr. Christoph Oing, Department of Oncology, Hematology and Bone Marrow Transplantation with Section of Pneumology, Mildred Scheel Cancer Career Center HaTriCs4, University Cancer Center Hamburg, Hamburg, Germany
BeWo	Choriocarcinoma	DMEM/F-12, GlutaMAX	10% FBS, 1% P/S (10,000 U), 1% L-Glutamin (200 mM)	ATCC, #CCL-98
JAR	Choriocarcinoma	DMEM (1x) + GlutaMAX-I	10% FBS, 1% P/S (10,000 U), 1% L-Glutamin (200 mM)	ATCC, #HTB-144
JEG-3	Choriocarcinoma	DMEM (1x) + GlutaMAX-I	10% FBS, 1% P/S (10,000 U), 1% L-Glutamin (200 mM)	ATCC, #HTB-36
NCCIT	Embryonal carcinoma	RPMI Medium 1640 (1x)	10% FBS, 1% P/S (10,000 U), 1% L-Glutamin (200 mM)	Dr. Christoph Oing, Department of Oncology, Hematology and Bone Marrow Transplantation with Division of Pneumology, Mildred Scheel Cancer Career Center HaTriCs4, University Cancer Center Hamburg, Hamburg, Germany
NT2/D1	Embryonal carcinoma	DMEM (1x) + GlutaMAX-I	10% FBS, 1% P/S (10,000 U), 1% L-Glutamin (200 mM)	Dr. Christoph Oing, Department of Oncology, Hematology and Bone Marrow Transplantation with Division of Pneumology, Mildred Scheel Cancer Career Center HaTriCs4, University Cancer Center Hamburg, Hamburg, Germany
TCam-2	Seminoma	RPMI Medium 1640 (1x)	10% FBS, 1% P/S (10,000 U), 1% L-Glutamin (200 mM)	Dr Janet Shipley, Division of Molecular Pathology, The Institute of Cancer Research, London, United Kingdom

### qRT-PCR analysis

For qRT-PCR analysis, RNA has been isolated, cDNA has been synthesized and PCR has been performed as described [13–15]. See Table 2 for oligonucleotides used in this study.

**Table 2:** Oligonucleotides used in this study

Gene	Forward primer	Reverse primer	Tm	Cycles
*ACTB*	CCATCATGAAGTGTGACGTGG	GTCCGCCTAGAAGCATTTGCG	60 °C	45
*CD9*	CCTGCTGTTCGGATTTAACTTCA	TGGTCTGAGAGTCGAATCGGA	60 °C	45
*CDX2*	GACGTGAGCATGTACCCTAGC	GCGTAGCCATTCCAGTCCT	60 °C	45
*ELF5*	TAGGGAACAAGGAATTTTTCGGG	GTACACTAACCTTCGGTCAACC	60 °C	45
*EOMES*	GTGCCCACGTCTACCTGTG	CCTGCCCTGTTTCGTAATGAT	60 °C	45
*GAPDH*	TGCCAAATATGATGACATCAAGAA	GGAGTGGGTGTCGCTGTTG	60 °C	45
*GATA3*	TCATTAAGCCCAAGCGAAGG	GTCCCCATTGGCATTCCTC	60 °C	45
*hCG*	AACACCCCTCACTCCCTGTCT	ATGCTCAGCAGCAGCAACA	60 °C	45
*HLA-G*	TTGCTGGCCTGGTTGTCCTT	TTGCCACTCAGTCCCACACAG	60 °C	45
*KRT7*	TGGAGGACTTCAAGAATAAGTACGAA	TCATGTAGGCAGCATCCACATC	60 °C	45
*MMP2*	TACAGGATCATTGGCTACACACC	GGTCACATCGCTCCAGACT	60 °C	45
*PLAA*	ACTGGCGAGTGTCTTGAAGTA	GCTGTTGTCACAAAGTCTCTACA	60 °C	45
*PRAME*	CGTAGACTCCTCCTCTCCCACAT	TGGGCGATATACTGCTCTTCCT	60 °C	45
*SOX17*	GATGCGGGATACGCCAGTGAC	GCTCTGCCTCCTCCACGAAG	60 °C	45
*SOX2*	ATGCACCGCTACGACGRGA	CTTTTGCACCCCTCCCATT	60 °C	45
*TEAD4*	TGATGCAGAGGGTGTATGGA	GATCAGCTCATTCCGACCAT	60 °C	45
*TFAP2C*	GGCCCAGCAACTGTGTAAAGA	GCAGTTCTGTATGTTCGTCTCCAA	60 °C	45

### Immunohistochemistry

For histopathological/immunohistochemical analysis SALL4, OCT3/4, D2-40, PRAME, SOX17, TFAP2c, GATA3, KRT7, EOMES, Sox2, hCG, GATA3, p63, SOX2 and CDX2 were tested. See Table 3 for antibodies used in this study. Immunohistochemistry was performed as described previously. Antigen retrieval was carried out at 97 °C in citrate buffer (pH 6) or EDTA buffer (pH 9). The following antibodies and dilutions were used: anti-SALL4 (monoclonal mouse, high buffer, 1:100, 30 min (min) of incubation, clone 6E3; CellMarque, Merck KGaA, Darmstadt, Germany), anti-CDX2 (monoclonal mouse, high buffer, ready to use, 25 min of incubation, clone DAK-CDX2; Dako, Agilent Technologies, Waldbronn, Germany), anti-GPC3 (monoclonal mouse, high buffer, ready to use, 30 min of incubation, clone IGI2; DCS Innovative Diagnostik-Systeme Dr. Christian Sartori GmbH & Co. KG, Hamburg, Germany), anti-desmin (monoclonal mouse, high buffer, ready to use, 20 min of incubation, clone D33; Dako), anti-carcinoembryonic antigen (CEA) (monoclonal mouse, high buffer, ready to use, 25 min of incubation, clone II7; Dako), anti-keratin (monoclonal mouse, high buffer, ready to use, 12.5 min of incubation, clone AE1/AE3; Dako), and anti-OCT3/4 (monoclonal mouse, high buffer, ready to use, 20 min of incubation, clone N1NK; Dako). The sections were incubated with a ready-to-use horseradish peroxidase-labelled secondary antibody at room temperature for 25 min (anti-rabbit/mouse, produced in goat; REAL EnVision Detection System; Dako, Agilent Technologies, Waldbronn, Germany). The substrate DAB + Chromogen system produces a brown end product and is applied to visualize the site of the target antigen (REAL DAB + Chromogen; Dako). Tissue samples were counterstained with Meyer's haematoxylin (Dako, Agilent Technologies, Waldbronn, Germany) for 8 min, and analyzed by the use of light microscopy.

**Table 3:** Used antibodies for immunohistochemical analysis

Antibodies	Company	Order No	Dilution	Application
SALL4	CellMarque	385 M	1/100	IHC
OCT3/4	Dako	IR09261-2	RTU	IHC
D2-40	Dako	IR072	RTU	IHC
SOX17	Sigma/Merck	ZRB1345	1/500	IHC
GATA3	CellMarque	390 M	390 M	IHC
SOX2	CellMarque	371R	RTU	IHC
hCG	Dako	IR50861-2	RTU	IHC
p63	Dako	IR66261-2	RTU	IHC
CDX-2	Dako	IR08061-2	RTU	IHC
GPC3	DCS	GI829R06	RTU	IHC
FOXA2	Invitrogen	MA5-15542	1:300	IHC

### In silico analyses

The GCT cohort (*n* = 149) in The Cancer Genome Atlas (TCGA) was analyzed by the cBioPortal [16]. A SEM cell character has been confirmed by hCG^+^ / SOX17^+^ / PRAME^+^ / SOX2^−^. The cohort has been screened for expression of trophoblast (differentiation) markers (*ABCG2*, *CD9*, *CDX2*, *DAB2*, *ELF5*, *ENPEP*, *EOMES*, *GATA2*, *GATA3*, *HAND1*, *hCG* (*CGA*), *HLA-G*, *KRT7*, *KRT19*, *MMP2*, *MMP9*, *PLAA*, *TACSTD2*, *TEAD4*, *TFAP2C* (also + in SEM), *VGLL1*).

## Results

### Patients

Patients were identified from the G3 Registry for SEM patients with hCG levels above normal [9]. Of *n* = 1031 patients with available data concerning their hCG levels at diagnosis screened, 39 patients (3.7%) showed hCG serum levels > 1000 U/l. FFPE tissues and RNA material was available for further analyses in seven and three cases, respectively. Within the cohort with tumor material available, the median pre-orchiectomy serum hCG level was 5356 U/l (range 1.224–40.909 U/L). All patients presented with a gonadal primary tumor at first diagnosis and UICC stage III in 5, Stage II in 4 and Stage I in 1 case, respectively. Thus, the tissue provided was material from orchiectomy specimens. Further patient characteristics of the patients with available tissue or RNA material are depicted in Table 4.

**Table 4:** Patient characteristics

Characteristics	Absolute number of patients (*n* = 10)
**Median serum hCG (U/L)**	53556 (range 1224–40909)
**UICC stage**	
I	1
II	4
III	5
IV	0
**IGCCCG classification**	
good prognosis	3
Intermediate prognosis	6
**First line therapy**	
cisplatin, etoposide, bleomycin (BEP)	6
cisplatin, etoposide (CE)	1
cisplatin, etoposide, ifosphamide (VIP)	1
Radiotherapy	1
**Average size of the tumor**	6.8 cm

UICC - Union for International Cancer Control; IGCCCG - International Germ Cell Cancer Cooperative Group

### Histomorphological and IHC analyses

**Fig. 1:**
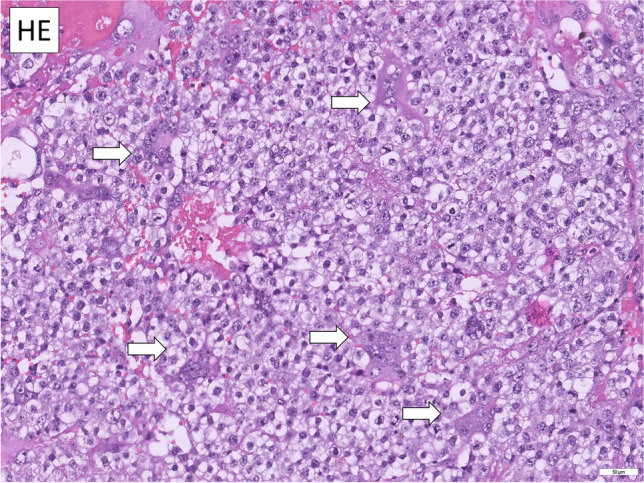
Histomorphological evaluation revealed tissue consistent with SEM and presence of syncytiotrophoblastic giant cells

**Fig. 2:**
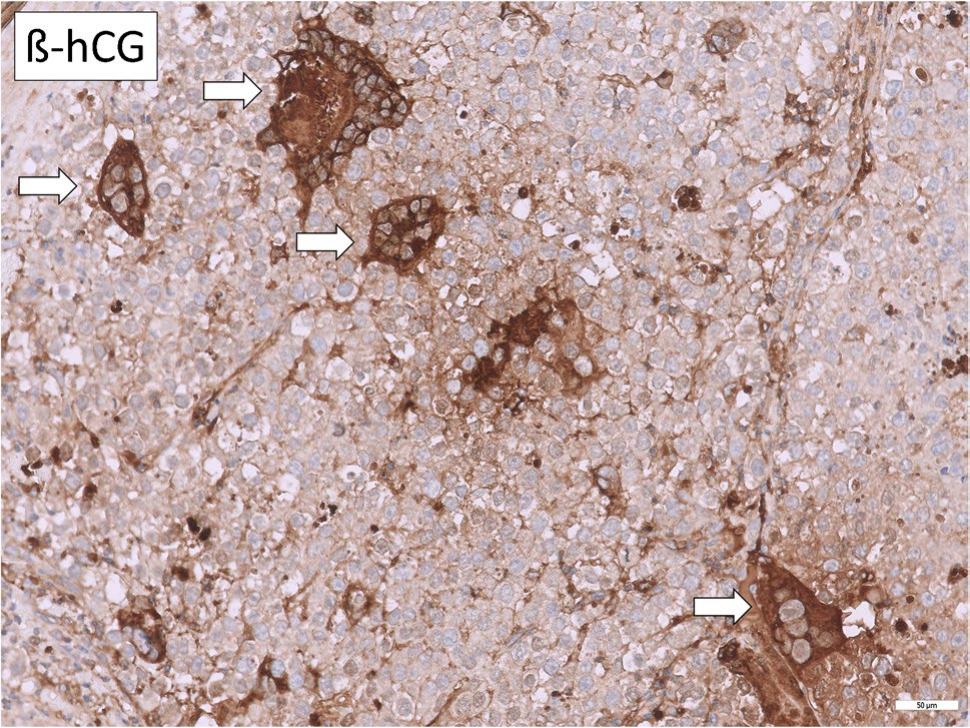
hCG was highly expressed in syncytiotrophoblastic giant cells in all 7 cases

**Fig. 3:**
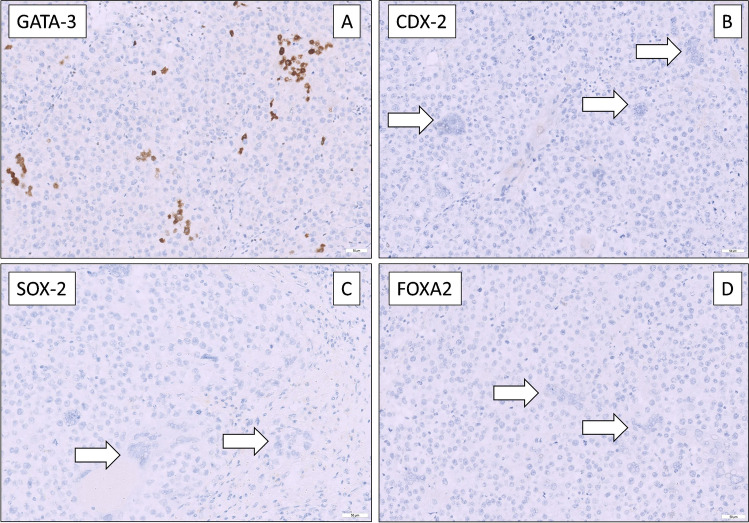
Immunhistochemistry revealed positivity for GATA 3 indicating the presence of synciotrophloblasts (**A**). Synciotrophoblasts were negative for CDX2 (**B**), SOX2 (**C**) and FOXA2 (**D**)

Hematoxylin and Eosin (HE) stained slides of the available FFPE tissues (*n* = 7) underwent central pathological review. Histomorphological evaluation revealed tissue consistent with SEM and presence of syncytiotrophoblastic giant cells as shown in Fig. 1 in all patients. No teratoma (TER) component could be found microscopically. For further tissue characterization and to rule out NSGCT components in the examined tissues, further IHC analysis were conducted. Here, hCG was highly positive in syncytiotrophoblastic giant cells in all 7 cases (Fig. 2), in accordance with hCG serum elevation in the investigated subset of patients. To prove trophoblastic nature of giant cells, IHC for GATA3 was conducted [17, 18], showing positivity in giant cells in all samples, but not the surrounding tumor tissue (Fig. 3A). Tissues were also stained for CDX2, SOX2 and FOXA2, which were negative [17] in accordance with the absence of yolk sac tumors (YST) and EC [19] (Fig. 3B–D). Thus, we analyzed a cohort of SEMs with trophoblastic populations, but no contribution of EC, YST or TER.

### Molecular analysis

**Fig. 4:**
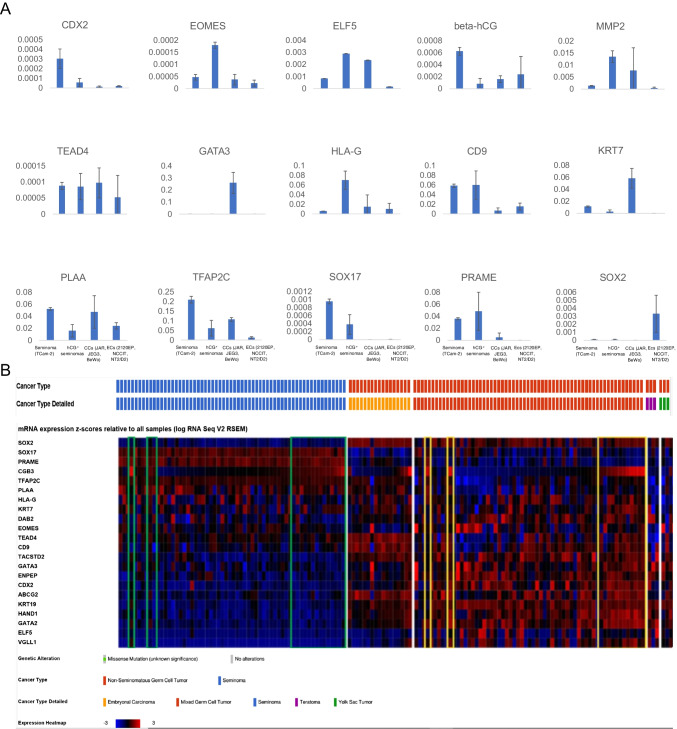
**A** qRT-PCR analysis of indicated trophoblast/CC marker genes in GCT cell lines (SEM: TCam-2; EC: 2102EP, NCCIT, NT2/D1; CC: JAR, JEG3, BeWo) and hCG + seminoma tissues. *SOX2*, *SOX17* and *PRAME* expression was analyzed to confirm a SEM cell character, while absent *SOX2* expression excludes EC sub-populations. **B** Screening of the TCGA GCT cohort (*n* = 149) for expression of seminoma (*SOX17*, *PRAME*), EC (*SOX2*) and trophoblast/CC marker genes. Green and yellow boxes highlight seminomas and EC with high *hCG* expression (mRNA z-score > 0.5), respectively

Next, we performed qRT-PCR analysis of trophoblast / CC markers of RNA from three SEM tissues with elevated hCG (hCG + SEMs) (Fig. 4). As controls, cell lines as proxies for SEM (TCam-2), EC (2102EP, NCCIT, NT2/D1) and CC (JAR, JEG3, BeWo) were included (Fig. 4A). Expression of *PRAME*, *SOX17* and absent expression of SOX2 again confirmed the seminomatous character of the hCG^+^ samples (Fig. 4A). qRT-PCR analysis detected trophoblast (stem cell) markers *CDX2*, *EOMES*, *ELF5, HLA-G, CD9, PLAA, TEAD4,* and *TFAP2C*, but not *GATA3* or *KRT7*, indicating a trophoblast / CC population within the analyzed SEM samples with elevated hCG (Fig. 4A).

### In silico analysis

Furthermore, we screened the GCT cohort from the TCGA (*n* = 149) for expression of trophoblast/CC markers (Fig. 4B). In *SOX17*^+^ / *PRAME*^+^ / *SOX2*^−^ SEM samples, 17 samples (28%) were identified with *hCG* expression (mRNA z-score > 0.5) (Fig. 4B, green boxes). In these samples, expression of only a few trophoblast markers was found (*CD9*, *KRT7*, *PLAA, TEAD4*, *TFAP2C*) (Fig. 4B). Interestingly, in mixed GCT with high hCG (yellow boxes), most of the analyzed marker genes were expressed (Fig. 4B).

## Discussion

Excessively elevated serum hCG at the time of diagnosis in SEM is an uncommon finding and therefore clinicians often suspect misdiagnosis of pure SEM in the presence of NSGCT components. As a testicular tumor with SEM and present NSGCT components is classified as NSGCT, this can imply prognostic and therapeutic consequences for the affected patients. In this study, we analyzed pure SEM with very elevated serum hCG levels over 1000 U/L, to confirm the diagnosis and find an explanation for high serum hCG levels in these cases. Elevated serum hCG in SEM with the presence of syncytiotrophoblasic giant cells is a known phenomenon, however, these cases usually appear with moderate serum hCG levels not exceeding 500 U/L. In this study, we analyzed tissue and RNA samples of SEM specimens in patients with serum hCG levels of up to 40,909 U/L. Central histomorphological analysis revealed typical findings of SEM in all patients and comprehensive IHC analysis proved a classical SEM-like phenotype with the presence of syncytiotrophoblastic giant cells. Moreover, an analysis of RNA of SEM from patients with high hCG levels revealed a typical gene expression signature of SEM with the presence of syncytiotrophoblastic giant cells. Importantly, since we could exclude presence of EC next to the trophoblast cells or within the SEM tissues in general, we hypothesize that SEM are able to undergo extra-embryonic differentiation into trophoblast/CC lineage, a concept already stated by Ulbright et al., but now strongly supported by our data [20].

Interestingly, our analysis of the TCGA data of mixed GCT with high *hCG* expression revealed that most of the analyzed trophoblast/CC marker genes were expressed, suggesting that either the CC component in hCG^+^ SEM tissues is too small to allow for detection of related markers in a bulk tumor tissue analysis or the trophoblastic / CC component in SEM is different than in NSGCT—a research question that might be addressed in the future.

An important limitation of this study is the relatively small cohort size of histologically and molecularly investigated SEM cases with very high serum hCG levels. The conclusion of the study, that all cases with exceptionally high hCG levels are secondary to the presence of syncitiotrophoblasts in seminoma is supported by a limited number of cases. Further validation is therefore needed. However, to our knowledge, this is the first study systematically reporting findings in tissue and RNA samples in this rare entity.

Taken together, our findings show that excessively increased hCG serum levels can appear in patients with pure SEM. To explain high hCG serum levels, it is important to diagnose the subtype of a SEM with syncytiotrophoblast giant cells. Due to the absence of EC, YST and TER, but presence of SEM and trophoblast/CC markers, we postulate that these hCG^+^ cells resemble trophoblast-like cells, which arose from extra-embryonic differentiation of SEM cells.

## Data Availability

The datasets generated during and/or analysed during the current study are available from the corresponding author on reasonable request.
